# biochem4j: Integrated and extensible biochemical knowledge through graph databases

**DOI:** 10.1371/journal.pone.0179130

**Published:** 2017-07-14

**Authors:** Neil Swainston, Riza Batista-Navarro, Pablo Carbonell, Paul D. Dobson, Mark Dunstan, Adrian J. Jervis, Maria Vinaixa, Alan R. Williams, Sophia Ananiadou, Jean-Loup Faulon, Pedro Mendes, Douglas B. Kell, Nigel S. Scrutton, Rainer Breitling

**Affiliations:** 1 Manchester Centre for Synthetic Biology of Fine and Specialty Chemicals (SYNBIOCHEM), Manchester Institute of Biotechnology, The University of Manchester, Manchester, United Kingdom; 2 School of Computer Science, The University of Manchester, Manchester, United Kingdom; 3 Institute of Systems and Synthetic Biology, University of Evry, Val d'Essonne, Evry, France; 4 School of Chemistry, The University of Manchester, Manchester, United Kingdom; 5 Center for Quantitative Medicine, UConn Health, 263 Farmington Avenue, Farmington, CT, United States of America; UCSD, UNITED STATES

## Abstract

Biologists and biochemists have at their disposal a number of excellent, publicly available data resources such as UniProt, KEGG, and NCBI Taxonomy, which catalogue biological entities. Despite the usefulness of these resources, they remain fundamentally unconnected. While links may appear between entries across these databases, users are typically only able to follow such links by manual browsing or through specialised workflows. Although many of the resources provide web-service interfaces for computational access, performing federated queries *across* databases remains a non-trivial but essential activity in interdisciplinary systems and synthetic biology programmes. What is needed are integrated repositories to catalogue both biological entities and–crucially–the relationships between them. Such a resource should be extensible, such that newly discovered relationships–for example, those between novel, synthetic enzymes and non-natural products–can be added over time. With the introduction of graph databases, the barrier to the rapid generation, extension and querying of such a resource has been lowered considerably. With a particular focus on metabolic engineering as an illustrative application domain, biochem4j, freely available at http://biochem4j.org, is introduced to provide an integrated, queryable database that warehouses chemical, reaction, enzyme and taxonomic data from a range of reliable resources. The biochem4j framework establishes a starting point for the flexible integration and exploitation of an ever-wider range of biological data sources, from public databases to laboratory-specific experimental datasets, for the benefit of systems biologists, biosystems engineers and the wider community of molecular biologists and biological chemists.

## Introduction

A detailed understanding of metabolism is critical to systems and synthetic biology approaches to biomedicine, drug discovery and development, and metabolic engineering for bioprocessing and industrial biotechnology. While biochem4j has a potentially broad appeal in a range of areas including fundamental physiology, evolution and understanding of pathogenicity, this initial work considers its application towards metabolic engineering. Within this context, a range of tasks, including designing synthetic pathways, collating organism-specific metabolic reconstructions, and interpreting metabolomics data, involves the drawing together of information from existing databases and ontologies. For example, the ChEBI ontology [1] catalogues chemicals and their relationships; KEGG provides a compendium of metabolic reactions and catalysing enzymes [2]; and NCBI Taxonomy describes the tree of life, providing an indication of how organisms (and therefore their enzymes) are related from an evolutionary perspective [3]. However, as yet there are few integrated data resources that covers all of these aspects [4], despite efforts having been made to incorporate such federated resources into workflow systems [5, 6]. Consequently making queries *across* these databases remains a major challenge.

Introduced here, biochem4j enables complex queries by linking numerous well-known and widely used chemical, biochemical and biology resources within a novel graph database. This implementation of a graph database not only provides a versatile resource for systems biologists and biosystems engineers, but also establishes the general concepts and techniques that will allow others to expand and modify the resource to meet their own requirements in a flexible manner. biochem4j offers an initial framework into which a wide variety of other data sources, including additional public databases and in-house experimental data, can now be incorporated.

**Fig 1:**
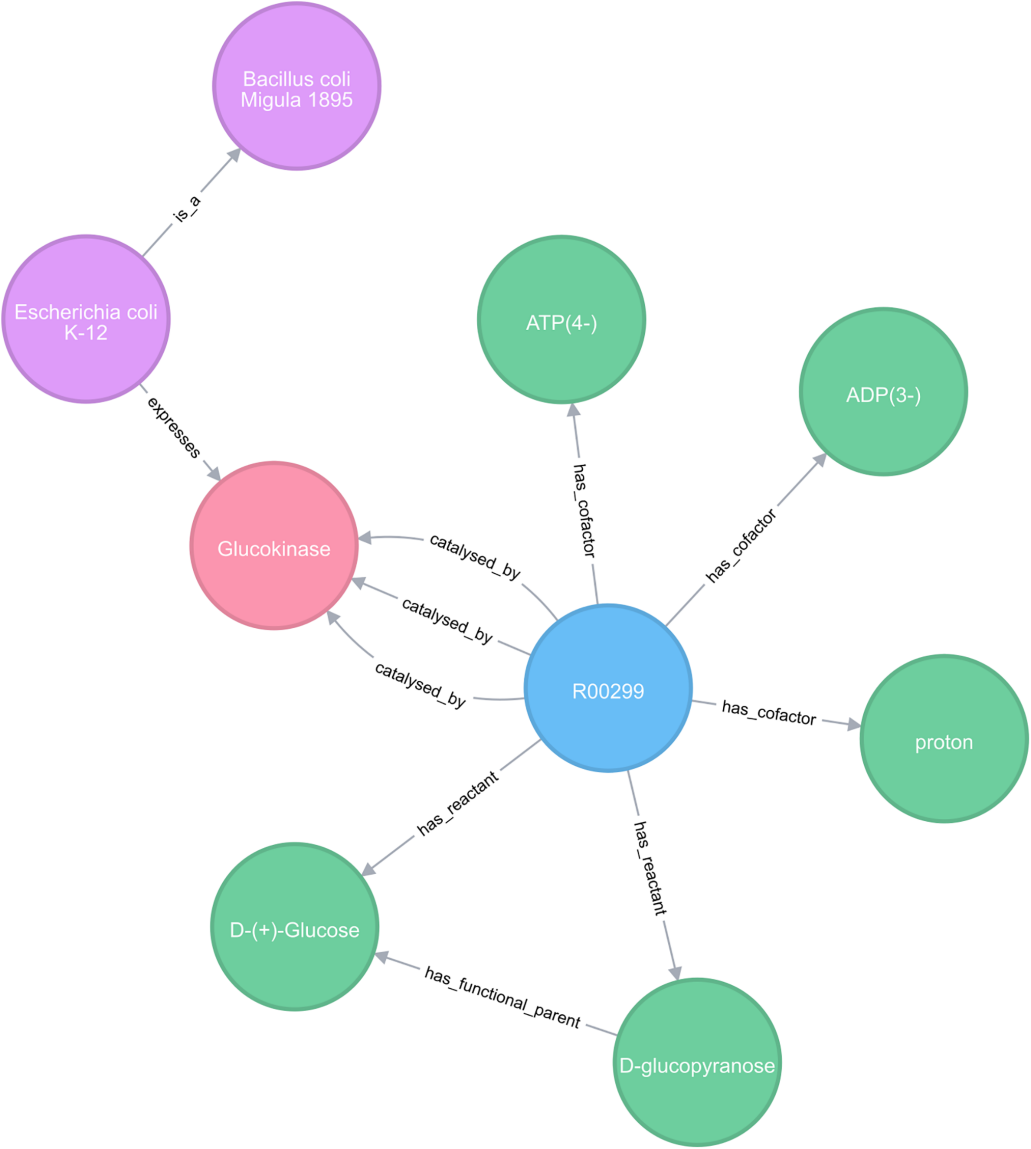
Example query output. Example output of the query investigating whether the reaction ATP:D-glucose 6-phosphotransferase occurs in *Escherichia coli* K-12. Neo4j provides a user-friendly web interface allowing relationships to be queried via CYPHER and results visually displayed as a graph that can be further explored interactively. Different types of data are shown (pink: Organism, red: Enzyme, blue: Reaction and green: Chemical). Relationships are displayed with their Types. In this example, the Organism Escherichia coli K-12 expresses the Enzyme Glucokinase, and Reaction R00299 has a relationship *catalysed_by* Glucokinase. Reaction R00299 *has_reactants* D-glycopyranose and D-glucopyranose-6-phosphate and *has_cofactors* ATP(4-) and ADP(3-). Additional relationships are shown: E. coli K-12 *is_a* Bacillus coli Migula 1895, and relationships between Chemicals are present, indicating that D-glucopyranose-6-phosphate *has_functional_parent* D-glucopyranose. This example indicates some of the relationships that exist between Organisms, Enzymes, Reactions and Chemicals, and an idea of the kind of queries that can be performed across them.

biochem4j is built on the freely available graph database, neo4j (Neo Technology, Inc., Malmö, Sweden; https://neo4j.com/) and is structured as follows. Organisms are represented and organised into their taxonomical tree based on information from the NCBI Taxonomy database. The organisms are linked to enzymes encoded in their genomes based on data extracted from UniProt [7]. Enzymes are linked to reactions that they catalyse using data from KEGG and Rhea [8]. Reaction definitions are extracted from MNXref [9], providing links to chemicals that participate in each reaction. Chemicals themselves are related to one another through data extracted from the ChEBI ontology. Specific entities of these data types (Taxonomy, Enzyme, Reaction, Chemical) are represented as nodes in the network and each node in the network contains metadata describing the entity. Thus, Chemicals contain terms such as molecular mass, and formula; Reactions contain Enzyme Classification (EC) numbers; enzymes hold UniProt IDs; and Organisms contain synonyms. (Full details are given in Table 1.) Nodes contain references to a number of external data resources, facilitating identifier mapping across resources. These nodes are connected through defined relationships. For example, Organisms contain *expresses* relationships towards Enzymes, and Reactions contain *has_reactant* relationships towards a Chemical. (Full details of all relationships are given in Table 2.) biochem4j therefore provides an integrated graph that allows complex queries to be performed across all of these linked nodes, along with a clear, interactive, visual representation of the network. (See Query 1 and Fig 1, below.)

**Table 1:** List of node types and their properties.

Label	Properties		
	Name	Description	Type
Organism	name	Name	String
	names	Synonyms	String[]
	taxonomy	NCBI Taxonomy id [3]	String
Enzyme	ec-code	Enzyme Classification id [16]	String
	entry	UniProt entry (e.g. HXKA_YEAST) [7]	String
	name	Name	String
	names	Synonyms	String[]
	uniprot	Uniprot id (e.g. P04806) [7]	String
Reaction	balance	Flag indicating whether the reaction is balanced	bool
	bigg.reaction	BiGG Models id [17]	String
	ec	Enzyme Classification id	String
	id	Unique id	String
	kegg.reaction	KEGG Reaction id	String
	metacyc	MetaCyc id [18]	String
	mnx	MNXref id [9]	String
	reactome	Reactome id [19]	String
	rhea	Rhea id [8]	String
	seed	SEED id [20]	String
	source	Source database for reaction data	String
Chemical	bigg.metabolite	BiGG Models id	String
	cas	CAS Registry Number (http://www.cas.org/content/chemical-substances)	String
	charge	Chemical charge	int
	chebi	ChEBI id [1]	String
	chemidplus	ChemIDplus id [21]	String
	chemspider	ChemSpider id [22]	String
	drugbank	DrugBank id [23]	String
	formula	Molecular formula	String
	hmdb	HMDB id [24]	String
	id	Unique id	String
	inchi	InChI string [25]	String
	kegg.compound	KEGG Compound id [2]	String
	kegg.drug	KEGG Drug id [2]	String
	kegg.glycan	KEGG Glycan id [2]	String
	knapsack	KNApSAcK id [26]	String
	lipidmaps	LIPID MAPS id [27]	String
	metacyc	MetaCyc id [18]	String
	mnx	MNXref id [9]	String
	molbase	Molbase id (http://www.molbase.com)	String
	monoisotopic_mass	Monoisotopic mass	float
	name	Name	String
	names	Synonyms	String[]
	pdb	Protein Data Bank id [28]	String
	pubmed	PubMed reference id	String
	reactome	Reactome id	String
	resid	RESID Database id [29]	String
	seed.compound	SEED id	String
	smiles	SMILES string	String
	source	Source database for chemical data	String
	umbbd.compound	University of Minnesota Biocatalysis/Biodegradation Database id [30]	String
	unipathway	UniPathway id [31]	String
	wikipedia.en	Wikipedia id	String

In biochem4j, nodes have a label (e.g. Chemical) and a number of associated properties, which can be used for both querying and in information retrieval. Not all nodes of a given label contain all of the available associated properties. For example, a generic chemical class such as “fatty acid” will not have a molecular mass, or a given reaction may not be represented in all source databases and therefore may not have a specific reference to Reactome.

**Table 2:** List of relationships between nodes.

From	To	Type	Properties		
			Name	Description	Type
Organism	Organism	is_a			
Organism	Enzyme	expresses	source	Source database	String
Reaction	Enzyme	catalysed_by	source	Source database	String
Reaction	Chemical	^1^has_reactant	stoichiometry	Stoichiometry of chemical reactant in reaction (negative for reactant, positive for product	int
		^1^has_cofactor	stoichiometry	Stoichiometry of chemical cofactor in reaction (negative for reactant, positive for product	int
Chemical	Chemical	^2^has_functional_parent			
		^2^has_parent_hydride			
		^2^has_part			
		^2^is_a			
		^2^is_conjugate_acid_of			
		^2^is_conjugate_base_of			
		^2^is_enantiomer_of			
		^2^is_substituent_group_from			
		^2^is_tautomer_of			

Such relationships can be considered to be “triples” in an analogous fashion to Resource Description Framework (RDF) representations. For example, “triples” exist from Reactions to Enzymes in the fashion “Reaction–catalysed_by–Enzyme”. Note that these relationships are all directional from the From label to the To label. ^1^Relationships between Reactions and Chemicals are either of the type “has_reactant” or “has_cofactor”. Cofactors are considered to be either low molecular mass compounds (less than 44 Da) or pairs of metabolites that are frequently occurring in the network (e.g. ATP and ADP). Frequency of occurance is calculated at the time of database loading (see source code: https://github.com/synbiochem/biochem4j). ^2^Chemical-to-Chemical relationships are extracted directly from the ChEBI database [1], and are described in https://www.ebi.ac.uk/training/online/course/chebi-quick-tour/what-chebi/chebi-ontology.

A key feature of graph databases is their flexible architecture, which supports customisation, extension and further development [10]. This distinguishes them from the relational databases that are currently routinely used to store and query biological databases [11]. As system requirements develop and additional data sources become available, it is trivial to add new data types and relationships to extend the graph database. The addition of these new types has no effect on the existing structure of the resource, and will not affect existing operations. In the context of biological databases such as biochem4j, this might mean, for example, that a graph database initially designed to store metabolic reactions could later easily be extended to also hold (and query) experimental postgenomic molecular profiling data without a wholesale redesign of the underlying schema. Similarly, it would also be trivial to extend the scope of the database to include encoding genome sequences, kinetic parameters and structural information linked to the enzyme data.

The use of graph databases in bio/cheminformatics is a recent development. Examples of existing work include a study into the use of graph databases and their application to substructure searching in cheminformatics [12], in the collation and analysis of individual biochemical pathways [13, 14], and in the storage of a genome scale model of human metabolism [15]. The ease with which disparate resources can be combined in a single integrated graph database means that it becomes considerably easier to perform queries and detect patterns across the whole range of available information.

Once collated, all of this information can then be explored and interpreted using a single uniform query interface. Therefore, biochem4j can act as a basic framework upon which more specialised (or more general) databases and applications can be built.

## Results

biochem4j links together data from a range of primary resources to provide a repository holding data on 1,544,257 named organisms, 2,457,504 enzymes, 36,765 reactions and 256,230 chemical species. These nodes are connected by 23,298,434 relationships, such as “Organism A *expresses* Enzyme X” or “Reaction Y *has_cofactor* Chemical Z”. Types of nodes and their properties are listed in Table 1. Relationships and their properties are available in Table 2. Details on the number of nodes and relationships are given in Table 3. The database can be updated through running a simple script, which is publicly available (see Methods).

**Table 3:** Numbers of nodes and relationships.

Node / relationship	Count	Percentage
Organism	1544257	
Organism–Organism	1544257	100
Organism–Enzyme	8431	0.546
Enzyme	2457504	
Enzyme–Organism	2454452	99.9
Enzyme–Reaction	2457504	100
Reaction	36765	
Reaction–Enzyme	12564	34.2
Reaction–Chemical	31296	85.1
Chemical	256230	
Chemical–Chemical	103075	40.2
Chemical–Reaction	19375	7.56

Counts of relationships are also given as percentages of nodes that have that relationship. For example, all (100%) Enzyme nodes have Enzyme-to-Reaction to relationships, while 34.2% of Reactions have Reaction-to-Enzyme relationships.

Like all neo4j databases, biochem4j can be queried through use of the CYPHER query language. It should be noted that this language is not immediately intuitive for novice users; we would not envisage that future applications built upon the biochem4j framework would require biologists to construct their queries directly in CYPHER. Instead, domain- and task-specific user interfaces would facilitate the interaction with the database, just as is the case for more conventional relational databases.

Due to its linking of taxonomical, enzyme, metabolic reaction and chemical data, a range of queries may be raised across all of these data types simultaneously. Concrete examples are given in Results, but more general queries that may be posed include:

- What are the known biochemical pathways from metabolite X to metabolite Y?
- What enzymes would be required to transform metabolite X to metabolite Y?
- Which organisms can perform pathway X?
- Which chemical transformations can metabolite X undergo?

The following query example illustrates the structure of the database, showing how chemical, reaction, enzyme and taxonomy data are related. The query asks, ‘does the reaction ATP:D-glucose 6-phosphotransferase (KEGG reaction R00299) occur in *Escherichia coli* K-12 (NCBI Taxonomy 83333)?’, essentially determining whether a link exists between the reaction node, an enzyme node, and the *E*. *coli* K-12 node in the database. Additionally, to demonstrate to connectivity between node types within the database, chemicals involved in the reaction, and the parent node of *E*. *coli* K-12 in the NCBI Taxonomy tree, are also returned. Note that implementing such a query across the individual databases used to populate biochem4j would be almost impossible using manual browsing–and would be computationally tedious even in an integrated relational database holding the same information. The results of the query are shown in Fig 1.

MATCH (c:Chemical)-[]-(r:Reaction {`kegg.reaction`: 'R00299'})-[]-(e:Enzyme)-[]-(child:Organism {taxonomy: '83333'})-[:is_a]->(parent:Organism)                 (1)

RETURN c, r, e, child, parent

Neo4j, by default, also includes a query-tuning tool. By prefixing a query with the term PROFILE, one can profile the query and retrieve execution times. In this instance the query ran in 411 ms.

A further useful feature of biochem4j is that the user interface, as shown in Fig 1, is interactive. Nodes can be expanded by clicking, so one may click on a Chemical node to see in which other Reactions the chemical participates, thus allowing the user to “walk” along the metabolic network in a stepwise fashion. Similarly, by clicking on a Reaction node, a synthetic biologist would quickly be able to determine how many homologous enzymes catalyse the reaction, and from which organisms do they originate.

Due to the broad scope of the CYPHER query language, far more complex queries can be written. Moreover, such queries can readily span multiple domains. The following examples of more complex queries, which integrate chemical, biochemical and taxonomical data, illustrate the power and flexibility of this approach, compared to conventional manual browsing of individual data sources.

To take an example from metabolic engineering, biochem4j can be used to perform a preliminary investigation into chemical space. One may wish, for example, to investigate the range of naturally occurring compounds belonging to a given chemical class. To return the name, chemical formula, ChEBI id and InChI string (a textual representation of chemical structure) of all known flavonoids, the following query can be run:

MATCH (parent:Chemical)<-[*]-(child:Chemical)

WHERE parent.name = 'flavonoid'                     (2)

AND EXISTS(child.formula)

RETURN DISTINCT child.name, child.formula, child.chebi, child.inchi

This query exploits the hierarchical chemical data held in biochem4j, which was extracted from the ChEBI ontology. The first two lines recursively select all descendent Chemical nodes under a parent Chemical node with name “flavonoid”. The third line limits the returned descendent Chemical nodes to those that have a chemical formula property. (This excludes Chemical nodes that do not have a specific chemical formula (or structure), but instead represent a generic chemical class, such as “flavans”.) The final line returns the unique set of chemicals by the requested fields. The query returns 1081 results in 4664 ms, which were exported in csv format and are available in S1 Appendix.

Considering the case of utilising biochem4j as an information source for either metabolomics data interpretation or method development, one can easily pose a query to the effect of “return all metabolites whose monoisotopic mass lies within a given mass range that are known to be present in reactions occurring in **all** *E*. *coli* strains” through the following syntax:

MATCH (c:Chemical)<-[:has_reactant]-(:Reaction)-[:catalysed_by]->(:Enzyme)<-[:expresses]-(:Organism)-[is_a]->(:Organism {taxonomy: '562'})            (3)

WHERE c.monoisotopic_mass > 400 AND c.monoisotopic_mass < 500

RETURN DISTINCT c.name, c.monoisotopic_mass, c.formula, c.charge

The first two lines of the query determine the structure of the query, linking Chemicals via Reactions and Enzymes to children of the top level *E*. *coli* Organism node in the taxonomical tree (NCBI Taxonomy: 562). The third line limits the returned Chemicals by a monoisotopic mass range. The final line specifies the Chemical data fields to return; in this example these are the name, monoisotopic mass, formula and charge. (The 111 results returned by this query in 1219 ms were exported in json format and are available in S2 Appendix.)

Of clear interest to the metabolic engineering community is the ability to use biochem4j in biosynthetic pathway design. The following query answers a typical question: what is the shortest path of metabolic reactions between a native (host) metabolite and a target molecule? (In this instance, the host is *E*. *coli* (taxonomy: 83333) and the target molecule pinocembrin (id: CHEBI:28157), a valuable flavonoid not naturally produced by this bacterium.)

MATCH (:Organism {taxonomy: '83333'})-->(:Enzyme)<--(:Reaction)-->(s:Chemical),

allShortestPaths((s)<-[r:has_reactant*1..8]->(t:Chemical {id: 'CHEBI:28157'}))

WITH s, r, t, range(0, size(r)-2) as idx                (4)

WHERE ALL (i IN idx

  WHERE r[i]['stoichiometry'] * r[i+1]['stoichiometry'] < 0)

RETURN s, r, t

The query is split into two parts. The first MATCH statement finds all chemicals that can be synthesised from enzymes expressed by *E*. *coli*. The second part finds the shortest path between each of these *E*. *coli* native metabolites and the target molecule. These are constrained such that (a) only *has_reactant* relationships between reactions and chemicals are considered, thus excluding “short cut” paths that include cofactors (see Methods); and (b) a maximum path length of 8 reactions is considered. A further consideration is to ensure that pathways are selected such that traversal through the graph moves from reactant to product. (This is encoded by the WHERE clause which considers reaction stoichiometry, which–as in constraint-based modelling–is defined as being negative for reactants and positive for products.) This query returns a number of pathways in 84 s, one of which was discovered by Fehér et al. using the pathway design tool RetroPath [32]; all alternative pathways are available in S1 Fig.

This example can be yet further extended, to take the collection of reactions that are non-native to the host, and return all enzymes (and their organisms) that catalyse each reaction:

MATCH (:Organism {taxonomy: '83333'})-->(:Enzyme)<--(:Reaction)-->(s:Chemical),

p = shortestPath((s)<-[r:has_reactant*1..8]->(t:Chemical {id: 'CHEBI:28157'}))

WITH p, s, r, t, range(0, size(r)-2) as idx

WHERE ALL (i IN idx                     (5)

  WHERE r[i]['stoichiometry'] * r[i+1]['stoichiometry'] < 0)

WITH [node in nodes(p) WHERE "Reaction" IN labels(node)] AS reactions

UNWIND reactions as reaction

MATCH (reaction)-[]->(enzyme:Enzyme)<-[]-(organism:Organism)

RETURN reaction, enzyme, organism

From this, an investigator could determine whether a single organism expresses enzymes that catalyse an entire pathway. Furthermore, through analysis of homologous enzymes catalysing each step, and the organisms that express them, a pathway engineer could determine which enzymes are expressed by thermophilic organisms, and are therefore amenable to industrial applications or to act as suitable candidates for directed evolution studies [33]. Similarly, related queries could investigate the metabolome of extremophile organisms, determining potential chemical targets or classes of chemical targets that may be produced under extreme conditions. Due to its structure, biochem4j is able to link taxonomical classification to enzyme and chemical space, enabling a range of queries of interest to the industrial biotechnology community.

## Discussion

The above examples give a flavour of potential queries that can be submitted to the biochem4j system, although the full range of queries is limited only by the user’s imagination. CYPHER is an extremely powerful (and continually developing) query language and as such, technically interested readers are encouraged to consult the CYPHER user guide for further details on the syntax and range of potential queries (https://neo4j.com/developer/cypher-query-language/). It is acknowledged that querying biochem4j directly with CYPHER may prove challenging for many biologists. However, the structure of the data model and the ease of development via the supplied neo4j web service should enable bioinformaticians to support wet-lab biologists through the development of specialised applications to support their desired queries.

A key difference between graph databases such as neo4j and RDF triple stores are in the way in which data is held internally. In RDF triple stores, statements (subject-predicate-object) typically specify objects that are primitives, such as integers, strings or floats. Considering the example of the Reaction to Enzyme relationship shown in Fig 1, a simple RDF representation would consist of the following triples:

<http://biochem4j.org/reaction/1>: has_name “R00299”

<http://biochem4j.org/reaction/1>: is_catalysed_by <http://biochem4j.org/enzyme/1>

<http://biochem4j.org/enzyme/1>: has_name “Glucokinase”

Whereas the neo4j equivalent, a single representation links two nodes that contain properties:

(a:Reaction {name: "R00299"})-[:catalysed_by]->(b:Enzyme {name: "Glucokinase"})

An RDF representation provides a collection of atomic statements, while graph databases specify relationships between more richly defined nodes, each of which being an individual database object. Such a representation increases ease of querying, an example of which is provided in S3 Appendix.

It is clear that a resource incorporating chemical, reaction, enzyme and taxonomic data will have applications in individual research fields that utilise these data types and the user may choose to query just one aspect of the resource. For example, metabolomics experts might consider chemical space, while evolutionary biologists might only have interest in the links between taxonomy and enzymes, ignoring the chemistry aspects of the system entirely. The text mining community may use the database as a source of synonyms for chemicals and enzymes. However, many fields, especially those based around metabolism, metabolic systems biology and industrial biotechnology, may wish to utilise all aspects of the resource simultaneously. Numerous queries, and applications, can therefore be built on top of biochem4j, primarily utilising the computer-readable web service interface that is provided by neo4j.

The simplicity of both querying and populating such graph database systems means that the user is free to extend the current system in whichever direction they wish. There is no limit to the additional node types and relationships that can be added to the system. Thus, extensibility into more bespoke data management solutions, including perhaps the storage of experimental data, is already supported.

From a synthetic biology perspective, a key consideration of biochem4j is this ability to grow over time. Current experimental approaches, not least the application of directed evolution to enzymes and exploitation of enzyme promiscuity, mean that such a repository needs to be able to handle the addition of novel enzymes and chemicals and the relationships between them. While existing enzymology resources such as BRENDA [34], ExplorEnz [35] and SABIO-RK [36] provide excellent coverage of wild type enzymology data (including results from a limited number of mutagenesis studies), there is still a requirement for an extensible repository that can handle the Big Data challenge that large-scale directed evolution studies provides. biochem4j in its current release primarily covers known, existing metabolic pathways, enzymes and naturally occurring metabolites. However, the structure is such that its extension into a more comprehensive synthetic biology repository, including large-scale enzyme variants libraries and their ability to produce non-naturally occurring metabolites, would be trivial to implement.

Further future directions of this work could also utilise the database as a source of mathematical models, with the ability to query for pathways and export results in standard modelling formats such as Systems Biology Markup Language (SBML) [37]. One could envisage exporting such models on different scales, including kinetic pathways of individual pathways [38], and genome-scale metabolic reconstructions [39]. Another potential area of interest would be a greater focus on metabolomics data analysis [40], with the integration of mass spectra of chemical compounds run under standard conditions. With the addition of such data, one could envisage a system exploiting existing chemical metadata, standardised mass spectra and metabolic network proximity to provide a comprehensive metabolite identification service.

The flexibility, extensibility and ease-of-use of graph database systems will provide enormous advantages in fast-moving areas such as biotechnological research over the coming years. biochem4j is an early example of their potential and can serve as a first step towards a family of related and interacting resources across a wide range of biological application domains.

## Methods

### Populating

biochem4j is built upon the freely available graph database, neo4j Community Edition, v3 (http://neo4j.com/), providing both a web and web service interface to the data.

biochem4j is populated through a number of custom Python modules. The basic workflow for each of the modules is to download and parse flat (text) files from source databases, generating nodes and relationships that are used to initialize a neo4j instance. The source databases are ChEBI, MNXref, Rhea, KEGG, UniProt and the NCBI Taxonomy. As the modules download the latest version of the flat files (or utilise APIs that manage versioning [41]), the content of biochem4j can be regularly updated as source databases expand in scope.

The key feature of the biochem4j is its integration of data. Such identifier mapping is a common problem in bioinformatics and has particular ramifications for the construction and merging of systems biology models [42, 43]. Therefore, an important aspect of the population process is to ensure that a given entity only appears once in the database. An example of this is with chemicals, which are present in both ChEBI and MNXref. In order to ensure uniqueness, properties of each node are mapped to a common namespace, which is defined by identifiers.org [44]. When a particular chemical entity is mapped to a KEGG entry in both ChEBI and MNXref, each source uses a different identifier to name the KEGG database (KEGG COMPOUND accession and kegg respectively). These are each mapped to the common identifiers.org namespace value, kegg.compound, allowing nodes representing the same entity but originating from different source databases to be merged. This allows a particular chemical species to maintain relationships in both the ChEBI ontology *and* also within the MNXref reaction network.

Another consideration of the biochem4j is in the specification of reaction definitions. In order to maximise the accuracy of the data and also to support future applications including constraint-based modelling [45], an attempt is made to balance all reaction data before submission. This makes use of an existing algorithm, ported from the SuBliMinaL Toolbox [46] which uses linear programming to balance reactions where possible, respecifying incorrect stoichiometries and adding commonly missing reactants such as protons and water.

All code is available through open source and the MIT License at https://github.com/synbiochem/biochem4j, with dependencies on core Python modules available from https://github.com/synbiochem/synbiochem-py.

### Deployment

The public version of biochem4j runs on the Google Compute Engine and is available at http://biochem4j.org. A Docker image is also available, allowing the system to be deployed on a large range of systems (https://www.docker.com/).

### Querying

Queries were performed through the default neo4j web interface using the query language CYPHER (http://neo4j.com/developer/cypher-query-language/). neo4j also supports a REST web service interface through which CYPHER queries may be POSTed to the database, with results returned in JSON format. A simple example (which returns 5 nodes selected at random) using curl, is as follows:

curl -H accept:application/json -H content-type:application/json \

  -d '{"statements":[{"statement": "MATCH (n) RETURN n LIMIT 5"}]}' \             (6)

    http://biochem4j.org/db/data/transaction/commit

Such web service calls allow for the support of biochem4j querying with established cheminformatics workflow packages such as Pipeline Pilot and KNIME [47].

## Supporting information

S1 Appendix Flavonoids (comma-separated). (CSV)

S2 Appendix E. coli metabolome (json format). (JSON)

S3 Appendix Comparison of neo4j / CYPHER with RDF / SPARQL. (PDF)

S1 Fig Pinocembrin pathway. (PNG)
