# Supplementary material for: biochem4j: Integrated and extensible biochemical knowledge through graph databases
Source: PLoS One. 2017 Jul 14;12(7):e0179130. doi: 10.1371/journal.pone.0179130 (PMC5510799; doi:10.1371/journal.pone.0179130)
Supplement: S3 Appendix — (PDF) [file pone.0179130.s003.pdf]

S3 Appendix: Comparison with RDF / SPARQL

The Resource Description Framework (RDF) provides another means for representing biochemical reactions as graphs. Popularly known as triple stores, RDF graphs store relationships in the form of subject-predicate-object triples: statements that often pertain to graph edges rather than graph structure. Thus, in storing biochemical reactions as RDF triples, a major consideration is the definition and design of the schema, i.e., ensuring that retrieving desired reaction information from the graph will not require an overly complicated query for matching graph edges. Defining a suitable RDF schema becomes a time-consuming bottleneck especially when aiming to federate diverse types of information, e.g., on small molecules, enzymes, organisms and reactions. In contrast, in neo4j, graphs are node-centric and queries are much more simple and intuitive. The example below shows the equivalent SPARQL (the language for querying RDF triple stores) query to query 3 in the manuscript.

```
PREFIX rdfs : <http ://www.w3.org/2000/01/rdf-schema#>
PREFIX rdf : <http://www.w3.org/1999/02/22-rdf-syntax-ns#>
PREFIX myschema : <http://myschema.org#>
SELECT ?name ?monoisotopic_mass ?formula ?charge
WHERE {
  ?c rdf:type myschema:Chemical .
  ?r rdf:type myschema:Reaction .
  ?e rdf:type myschema:Enzyme .
  ?o rdf:type myschema:Organism .
  ?c myschema:Chemical:hasName ?name ;
     myschema:Chemical:hasMonoisotopicMass ?monoisotopic_mass ;
     myschema:Chemical:hasFormula ?formula ;
     myschema:Chemical:hasCharge ?charge .
  FILTER (?monoisotopic_mass > 400 && ?monoisotopic_mass < 500) .
  ?r ?someRelation1 ?c ;
     ?someRelation2 ?e .
  ?e ?someRelation3 ?o .
  ?o myschema:Organism:taxonomy “562” .
}
```
