# Supplementary figures and images for: biochem4j: Integrated and extensible biochemical knowledge through graph databases
Source: PLoS One. 2017 Jul 14;12(7):e0179130. doi: 10.1371/journal.pone.0179130 (PMC5510799; doi:10.1371/journal.pone.0179130)

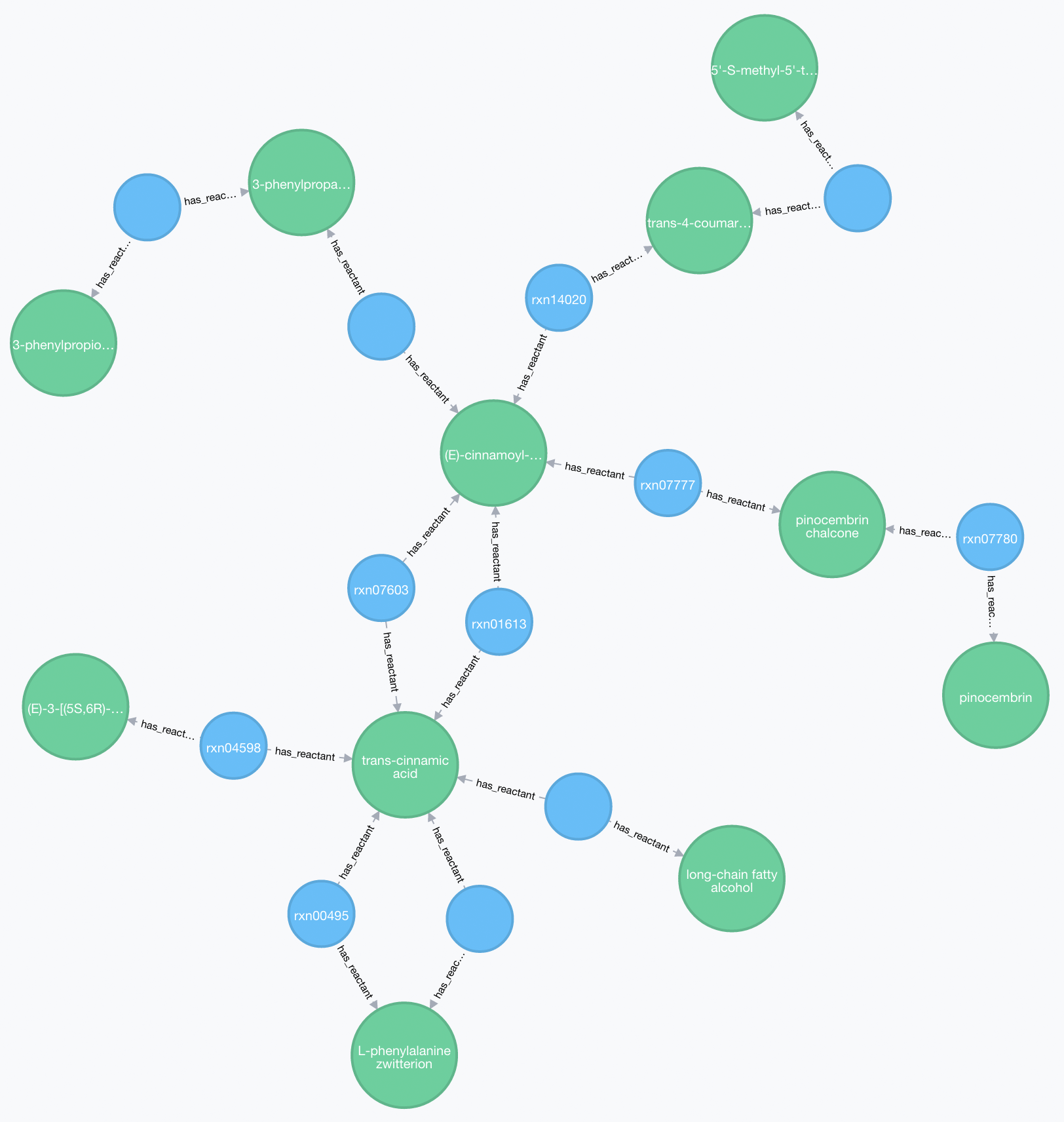

Supplement: S1 Fig — (PNG) [file pone.0179130.s004.png]
